# Competitive Binding of Ozanimod and Other Sphingosine 1-Phosphate Receptor Modulators at Receptor Subtypes 1 and 5

**DOI:** 10.3389/fphar.2022.892097

**Published:** 2022-06-17

**Authors:** Julie V. Selkirk, Andrea Bortolato, Yingzhuo Grace Yan, Nathan Ching, Richard Hargreaves

**Affiliations:** ^1^ Neuroscience Thematic Research Center, Bristol Myers Squibb, Princeton, NJ, United States; ^2^ Molecular Structure & Design, Bristol Myers Squibb, Princeton, NJ, United States

**Keywords:** G protein-coupled receptor, multiple sclerosis, orthosteric binding site, radioligand binding, sphingosine 1-phosphate

## Abstract

Ozanimod, a sphingosine 1-phosphate (S1P) receptor modulator, binds with high affinity selectively to S1P receptor subtypes 1 (S1P_1_) and 5 (S1P_5_), and is approved in multiple countries for treating adults with relapsing forms of multiple sclerosis (MS) or moderately to severely active ulcerative colitis (UC). Other S1P receptor modulators have been approved for the treatment of MS or are in clinical development for MS or UC, but it is unknown whether these compounds bind competitively with each other to S1P_1_ or S1P_5_. We developed a competitive radioligand binding assay using tritiated ozanimod and demonstrate full displacement of ozanimod by S1P (endogenous ligand), suggesting that ozanimod binds to the S1P_1_ and S1P_5_ orthosteric binding sites. S1P receptor modulators FTY720-p, siponimod, etrasimod, ponesimod, KRP-203-p, and amiselimod-p also completely displacing radiolabeled ozanimod; thus, on a macroscopic level, all bind to the same site. Molecular docking studies support these results and predict the binding of each molecule to the orthosteric site of the receptors, creating similar interactions within S1P_1_ and S1P_5_. The absolute free energy perturbation method further validated key proposed binding modes. Functional potency tightly aligned with binding affinities across S1P_1_ and S1P_5_ and all compounds elicited S1P_1_-mediated β-arrestin recruitment. Since all the S1P modulators included in this study display similar receptor pharmacology and compete for binding at the same site, they can be considered interchangeable with one another. The choice of any one particular agent should therefore be made on the basis of overall therapeutic profile, and patients can be offered the opportunity to switch S1P medications without the potential concern of additive S1P pharmacology.

## Introduction

Sphingosine 1-phosphate (S1P) is an important lipid signaling molecule present in the systemic circulation that mediates its effects through the binding and activation of a family of five G protein–coupled receptor (GPCR) subtypes, referred to as S1P_1_ through S1P_5_, which regulate many fundamental biological processes through their diverse cellular and tissue expression profiles (Brinkmann, 2007; Cohan et al., 2020). A key role of S1P is the modulation of immune cell function, and S1P_1_ has been shown to be an important receptor for the trafficking of lymphocytes out of the lymphoid tissue following the S1P concentration gradient into the blood stream and then circulating to the target tissue to mount an immune response (Brinkmann et al., 2004; Thangada et al., 2010). Agonism of S1P_1_ results in rapid down-modulation of cell surface receptors (Graler and Goetzl, 2004; Scott et al., 2016), thus rendering the lymphocytes incapable of sensing the S1P gradient, resulting in retention of the cells in the lymphoid tissue and reduced levels in the systemic circulation (Harris et al., 2020). As such, S1P_1_ has served as an attractive receptor for pharmacological intervention in autoimmune diseases, and S1P receptor modulators have been studied clinically in diseases and conditions such as organ transplant rejection, lupus, atopic dermatitis, inflammatory bowel disease, and multiple sclerosis (MS) (Park and Im, 2017; Tanaka et al., 2020; Roy et al., 2021).

The first S1P receptor modulator to successfully obtain marketing authorization was fingolimod (FTY720), a nonselective S1P receptor modulator that has activity at S1P_1_, S1P_3_, S1P_4_, and S1P_5_, for the treatment of relapsing and remitting MS in adult patients (Brinkmann et al., 2002; Gilenya approval language, 2019). Due to the nonselective nature of fingolimod’s interaction with the S1P receptors, target engagement on non-immune cell types may lead to documented adverse safety findings, such as hemodynamic and ocular effects (Kappos et al., 2010; Calabresi et al., 2014). Hence, selectivity was further fine-tuned with second-generation molecules to maximize benefits and lessen the risk of side effects, leading to the development of the S1P_1_ and S1P_5_ selective modulators siponimod and ozanimod, which have also been approved for relapsing forms of MS (Mayzent approval language, 2021; Zeposia approval language, 2021; Roy et al., 2021). In addition, ozanimod has demonstrated significant efficacy in the clinical treatment of ulcerative colitis (UC) (Sandborn et al., 2021) and recently received marketing approval for this second indication (Zeposia approval language, 2021).

Several S1P receptor modulators are either clinically available or in development for the treatment of various autoimmune disorders, and as such, there will be further extended choices in the future for patients and physicians as to which medication might be optimal for a given indication. The purpose of the study presented here was to determine if these key S1P receptor modulators bind to the same binding site within S1P_1_ and S1P_5_ and could, therefore, be considered pharmacologically similar in their mode of binding action. This information, together with the overall efficacy and safety profile of the various molecules, is important as health care providers may consider switching patients to newer S1P receptor modulators, such as ozanimod, given their competitive binding within the same site. We have utilized a radiolabeled form of ozanimod to conduct this assessment, and because ozanimod is selective for human S1P_1_ and S1P_5_ (Surapaneni et al., 2021), our binding assessment is limited to these subtypes. To determine the correlation of the receptor binding affinity to the functional potency of S1P_1/5_ activation, we also conducted G protein–coupling assays and included assessment across all five human S1P receptor subtypes to provide a thorough head-to-head comparison of selectivity across the approved and investigational modulators.

## Materials and Methods

### Materials

Ozanimod was synthesized at Celgene/BMS Science Park (San Diego, California, United States). S1P was purchased from Enzo Life Sciences (Farmingdale, New York, United States), FTY720-phosphate (FTY720-p) was obtained from Toronto Research (North York, Ontario, Canada), and siponimod was acquired from Fisher Scientific (Pittsburgh, Pennsylvania, United States). Amiselimod was custom synthesized by Chemveda (San Diego, California, United States), KRP-203-phosphate (KRP-203-p) by SD Chem (San Diego, California, United States), and etrasimod and ponesimod by PharmaBlock (Hatfield, Pennsylvania, United States). The radioligand, tritium-labeled ozanimod ([^3^H]-ozanimod) was custom synthesized by Novandi Chemistry (Södertälje, Sweden) and [^35^S]-guanosine-5'-(γ-thio)-triphosphate ([^35^S]-GTPγS) was purchased from Perkin Elmer (Waltham, Massachusetts, United States). Guanosine diphosphate (GDP; catalog number G7127) and all other buffer reagents were purchased from Sigma Aldrich (St. Louis, Missouri, United States).

### Cell Membrane Preparation

Stable clones of Chinese hamster ovary (CHO) cells expressing human S1P_1_ and S1P_5_ were generated by transfecting the cells with the N-terminal hemagglutinin-tagged receptor in pcDNA3.1 expression vectors followed by antibiotic selection for S1P_1_ and S1P_5_ and picking of clones using cyclic adenosine monophosphate assay, fluorescence-activated flow cytometry using the associated tag as well as functional [^35^S]-GTPγS binding assays. Stable clones of CHO cells expressing human S1P_2_, S1P_3_, and S1P_4_ with an N-terminal FLAG tag were purchased from Multispan, Inc. (Hayward, CA, United States; catalog numbers C1051-1, CG1049-1, and CG1052-1 for S1P_2_, S1P_3_, and S1P_4_, respectively). Cells were grown to confluence in adherent culture in 500-cm^2^ culture trays before being detached with cell scraper in cell-lifting buffer (10 mM HEPES/154 mM NaCl/6.85 mM ethylenediaminetetraacetic acid [EDTA], pH 7.4) and pelleted by centrifugation for 5 min at 1000 rpm. Cell pellets were then resuspended and homogenized in membrane preparation buffer (10 mM HEPES/10 mM EDTA, pH 7.4) using a Polytron PT 1200E homogenizer (Kinematica, Luzern, Switzerland). To collect the membrane pellet, cell homogenates were centrifuged at 48,000 × *g* at 4°C for 30 min. After discarding the supernatant, the pellet was rehomogenized and recentrifuged as described above. The final pellet was collected and homogenized in ice-cold resuspension buffer (10 mM HEPES and 0.1 mM EDTA, pH 7.4). Aliquots of samples were stored at −80°C until needed for [^3^H]-ozanimod radioligand binding or [^35^S]-GTPγS binding assays.

### Saturation Radioligand Binding

Saturation binding assays were performed in 96-well Optiplate-96HB plates (Perkin Elmer, catalog number 6005500) in a final volume of 200 μl. One assay plate was prepared each for CHO-human S1P_1_, CHO-human S1P_5_, and CHO-K1 parental cells. For determination of total binding, half of the assay plate was prepared with 60 μl/well of 0.33% DMSO control, and the other half of the plate was prepared for nonspecific binding measurements with 60 μl/well of 33.33 μM unlabeled ozanimod. Serial dilutions of [^3^H]-ozanimod, from 200 to 0.6625 nM, were performed in assay buffer (20 mM HEPES [pH 7.4], 10 mM MgCl_2_, 100 mM NaCl, 1 mM EDTA, 0.1% fatty acid–free BSA, and 30 μg/ml saponin) using glass vials. Each concentration of radioligand (40 μl/well) was then added in triplicate to the total binding and nonspecific binding wells. The reaction was started with the addition of 100 μl of membrane preparations to each well to yield a final protein concentration of 4.8 μg/well. Plates were sealed and incubated at room temperature with gentle agitation for 60 min before assay termination by filtration.

Filter plates were prepared by adding 80 μl/well of 0.3% poly (ethyleneimine) and incubating for 60 min at room temperature before washing with 150 ml of filtration buffer (50 mM Tris HCl [pH 7.4], 5 mM MgCl_2_, and 1 mM EDTA) using a FilterMate-96 harvester. After filtration, the excess unbound radioligand was washed with 10 × 200 μl/well wash cycles using filtration buffer plus an additional prolonged wash with 500 ml of total filtration buffer per plate. Filter plates were then allowed to air dry before the addition of 50 μL/well of MicroScint-20 cocktail, plate sealing, and reading on a MicroBeta2 microplate scintillation counter. To quantify free [^3^H]-ozanimod, 40 μl of each concentration was added directly to a filter plate in triplicate and air dried, and 50 μl/well of MicroScint-20 cocktail was added before the plate was sealed and read.

Raw counts of 60 s per well were collected from the MicroBeta2 counter. The binding of [^3^H]-ozanimod to human S1P_1_ or S1P_5_ or parental CHO membranes was calculated by subtracting the nonspecific binding (that which bound in the presence of 10 μM unlabeled ozanimod) from the total binding. The specific binding of [^3^H]-ozanimod to human S1P_1_ or S1P_5_ was determined by subtracting out the binding to the parental CHO membranes to calculate the *K*_*D*_ values for [^3^H]-ozanimod to S1P_1_ and S1P_5_.

### Competition Radioligand Binding

Competition radioligand binding assays with [^3^H]-ozanimod were also performed in 96-well Optiplate-96HB plates (Perkin Elmer, catalog number 6005500) in a final volume of 200 μL S1P was prepared by resuspension in methanol to a concentration of 1 mM, sonication for 1 h in a 37°C water bath to dissolve, and, once dissolved, pipetting into 100 μL (100 nmol) aliquots, and the solvent was evaporated over a stream of nitrogen gas. S1P working stock was initially prepared to a 400 μM solution in 10 mM Na_2_CO_3_/2% β-cyclodextrin from an individual 100 nmol aliquot and then serial diluted in assay buffer (20 mM HEPES/10 mM MgCl_2_/100 mM NaCl/1 mM EDTA, 0.1% fatty acid–free bovine serum albumin [BSA], and 30 μg/ml saponin) and 60 μl/well transferred to assay plates. Test compounds in 100% dimethyl sulfoxide (DMSO) were serial diluted in DMSO directly into the assay plates using the Tecan D300E digital printer (Tecan, Männedorf, Switzerland) in a total volume of 0.4 μl followed by either 60 μl/well of assay buffer or 60 μl/well 3.33× of unlabeled ozanimod (0.3 μM final for S1P_1_ or 3 μM for S1P_5_) to define the nonspecific binding. Next, 40 μl/well of 5× [^3^H]-ozanimod (3 nM final for S1P_1_ or 5 nM final for S1P_5_) was added before the experiment was initiated by the addition of 100 μl membranes to all wells to yield a final protein concentration of 4.8 μg/well. Assay plates were sealed and incubated at room temperature with gentle agitation for 60 min before filtration as described above.

Data were again analyzed as raw counts per 60 s per well collected from the MicroBeta2 counter. Raw data were normalized to the DMSO vehicle (0% inhibition of [^3^H]-ozanimod binding) and 0.3 μM (S1P_1_) or 3 μM (S1P_5_) ozanimod (100% inhibition of [^3^H]-ozanimod binding). For the inhibition of [^3^H]-ozanimod binding by test compound, concentration response curves were analyzed with GraphPad Prism version 8.0.0 (GraphPad Software, San Diego, California, United States) using nonlinear regression and one site fit to generate *K*_*i*_ values per the predetermined *K*_*D*_ values for [^3^H]-ozanimod.

### [^35^S]-GTPγS Binding

To quantitate receptor activation and G protein coupling, [^35^S]-GTPγS binding assays were performed in 96-well nonbinding surface plates (Corning, New York, New York, United States; catalog number 3604) in a final volume of 200 μl. Test compounds were serial diluted in DMSO directly to the assay plate using the Tecan D300E digital printer in a total volume of 0.4 μl. The endogenous ligand, S1P, was used as a normalization control and was separately prepared as a 400 μM stock solution from a 100 nmol S1P pellet in 10 mM Na_2_CO_3_/2% β-cyclodextrin. The serial dilution of S1P was performed by hand in assay buffer (20 mM HEPES/10 mM MgCl_2_/100 mM NaCl/1 mM EDTA, 0.1% fatty acid–free bovine serum albumin [BSA], and 30 μg/ml saponin), and 40 μl/well was transferred to wells containing 0.4 μl of DMSO vehicle. All wells were brought to a total volume of 40 μl with assay buffer, and the reaction was initiated by the addition of 120 μl/well of assay buffer containing a mixture of 40 μg/ml S1P_1_–S1P_5_ membranes, 5–50 μM GDP (5 μM for S1P_2_ and S1P_4_, 16.67 μM GDP for S1P_1_ and S1P_5_, and 50 μM for S1P_3_), and 2.5 mg/ml of WGA PVT SPA beads (Perkin Elmer catalog number RPNQ0001). Assay plates were sealed and incubated at room temperature with gentle agitation for 30 min before adding 40 μl/well of 5× [^35^S]-GTPγS (200 p.m. final) (Perkin Elmer, catalog number NEG030X250UC) in basic assay buffer (20 mM HEPES/10 mM MgCl_2_/100 mM NaCl/1 mM EDTA, pH 7.4) and then plates were resealed for an additional 40-min incubation at room temperature with gentle agitation. The experiment was terminated by centrifugation of the plates at 1000 rpm for 3 min using an Eppendorf (Hamburg, Germany) 5810R centrifuge and read on the MicroBeta2 microplate scintillation counter. Data were analyzed as raw counts per 40 s per well as collected from the MicroBeta2 counter. Raw counts were analyzed with GraphPad Prism version 8.0.0 using nonlinear regression to generate concentration response curves. Data were normalized to the percentage response relative to the internal S1P control (maximal S1P response was considered 100%, and the S1P basal response was considered 0%). The potency was measured as the concentration required to elicit a 50% response (EC_50_), and the magnitude of the test compound response, or intrinsic activity, was calculated as the difference between the maximum and the minimum of each independent agonist concentration response curve.

### S1P_1_-Mediated β-arrestin Recruitment Assay

Human S1P_1_ β-arrestin assays were carried out using the PathHunter® eXpress EDG1 CHO-K1 β-arrestin GPCR Assay (DiscoverX, Cat# 93-0207E2, including frozen cell stock, assay media, and assay plate). EDG1(S1P_1_)/β-arrestin CHO-K1 cells were thawed by adding 0.5 ml warm media to the vial. The cells were then transferred to 11.5 ml warm media, seeded at a density of 10,000 cells per well in a 96 well assay plate in a volume of 100 μL per well and incubated overnight in a 37°C CO_2_ incubator. On the day of assay, S1P was manually serially diluted in media containing 3.33% DMSO in a 96 well V-bottom plate (Corning, Cat#3363) and the test compounds were serially diluted in DMSO directly into the same 96 well plate containing 50 μl/well assay media using the Tecan D300E digital printer to prepare 11X compound solutions containing 3.33% DMSO final. Compounds were then added to the EDG1(S1P_1_)/β-arrestin CHO-K1 cells—10 μl per well of the 11X stock was added to the 100 ul per well of cells and the plate was incubated for 90 min in a 37°C CO_2_ incubator. The detection solution was prepared according to the manufacture’s protocol and 55 μl was added to each well of assay plate. The plate was then incubated for 1 h at room temperature before being read on a SpectraMax5 plate reader. Basal β-arrestin recruitment was determined using medium containing DMSO alone.

### Computational Chemistry

The 3D coordinates of human S1P_1_ were downloaded as described by Burley et al. (2019) from the Protein Data Bank ID 3V2Y (Hanson et al., 2012). The human S1P_5_ protein sequence was downloaded from UniProt (ID Q9H228). The homology model of human S1P_5_ was created in Prime using methods described by Jacobson et al. (2004) using human S1P_1_ as the template. Siponimod, FTY720-p, ozanimod, ponesimod, etrasimod, S1P, amiselimod-p, and KRP-203-p chemical structures were drawn in Maestro 2020-4 (Schrödinger, LLC, New York, NY, United States) and prepared for docking using LigPrep (Schrödinger), and custom force field parameters were optimized using the Force Field Builder (Schrödinger). Ozanimod was docked in the homology model using Glide SP (Halgren et al., 2004). The resulting system was prepared for molecular dynamics (MD) simulation using System Builder in Maestro. The protocol embedded the complex in a POPC (1-palmitoyl-2-oleoyl-*sn*-glycero-3-phosphocholine) membrane bilayer; it added equilibrated water molecules in the simulation box and neutralized the total charge, adding the correct type and number of ions. The system was equilibrated using Desmond MD software 2020 (Desmond Molecular Dynamics System, D. E. Shaw Research, New York, NY, United States) using the default protocol for membrane systems plus an additional 50 ns MD simulation (NVT ensemble at 300 K temperature). After removal of ozanimod, the equilibrated system was used to dock the other ligands using Glide SP. To further validate the predicted poses, the absolute free energy of binding of selected ligands were evaluated using the absolute free energy perturbation (AFEP+) method in Maestro 2020-4 using default settings (Wang et al., 2015).

## Results

### Radioligand Binding

**FIGURE 1:**
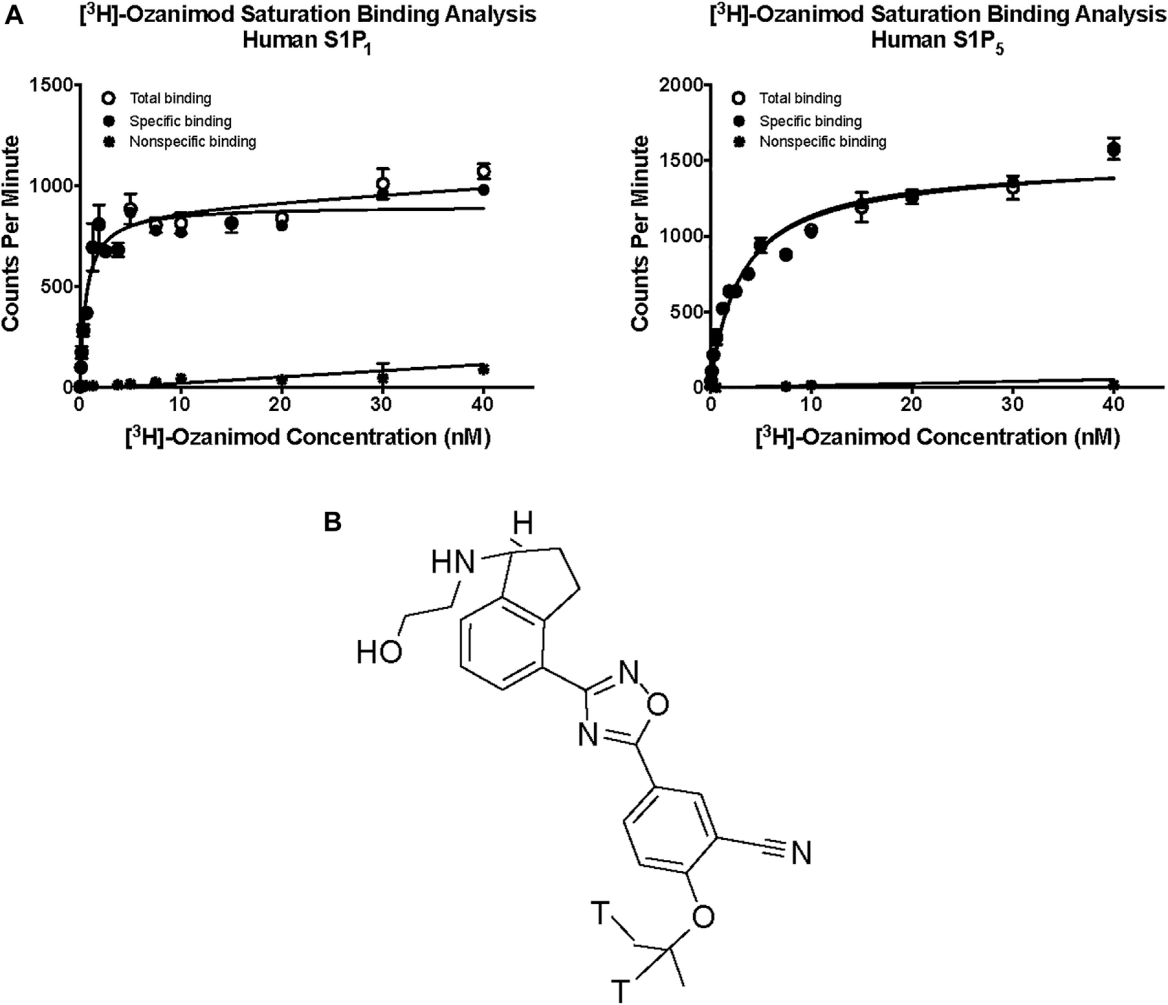
**(A)** Saturation binding analysis for [^3^H]-ozanimod to human S1P_1_ and human S1P_5_. **(B)** Structure of [3H] ozanimod. S1P1, sphingosine 1-phosphate receptor subtype 1; S1P5, sphingosine 1-phosphate receptor subtype 5.

**FIGURE 2:**
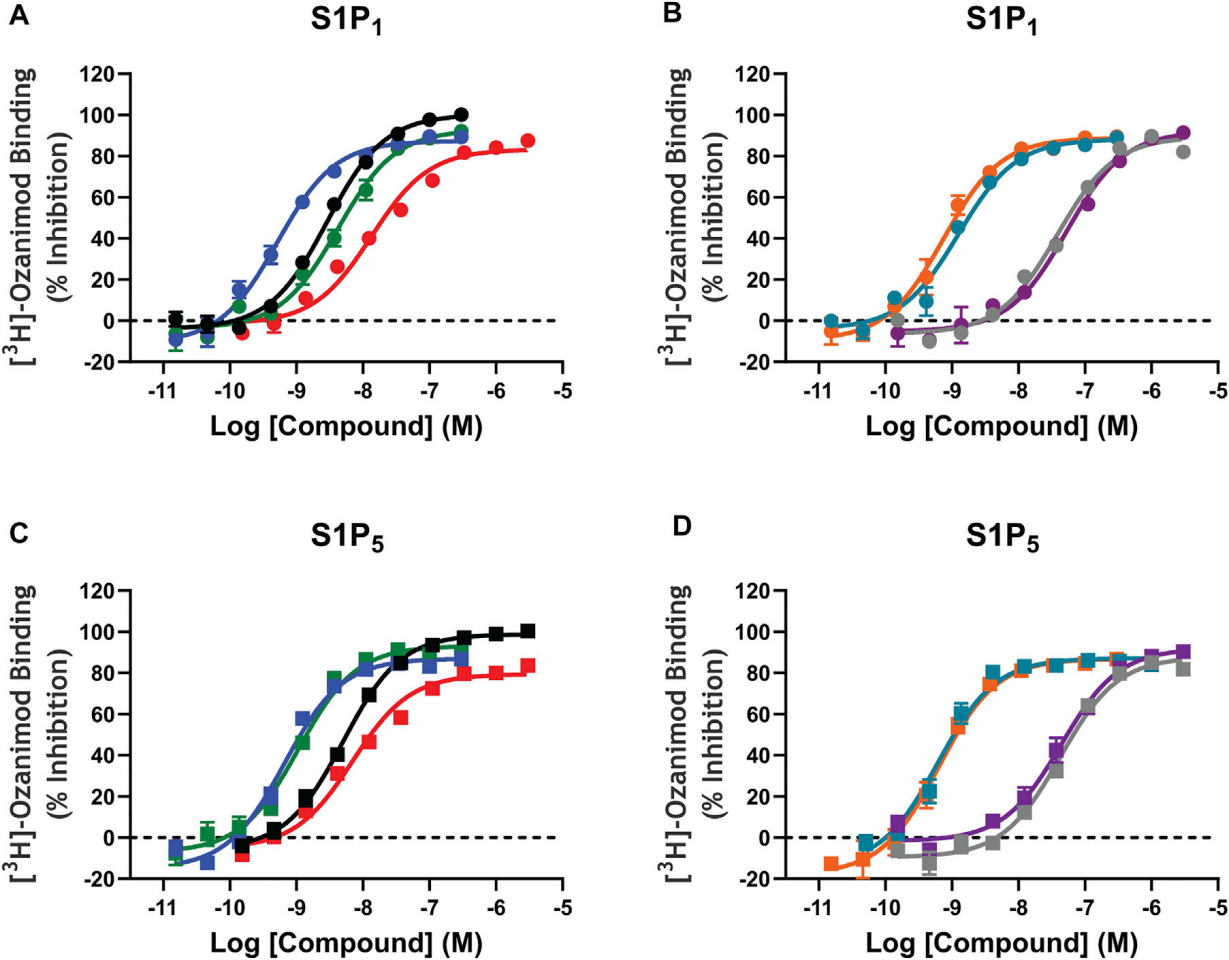
[^3^H]-Ozanimod competition radioligand binding concentration response curves. **A–D:** [^3^H]-ozanimod was competitively displaced with increasing concertation of ozanimod (black curves), fingolimod-p (blue curves), siponimod (green curves), S1P (red curves), etrasimod (grey curves), ponesimod (purple curves), KRP-203-p (teal curves), or amiselimod-p (orange curves) using Chinese hamster ovary cell membranes stably expressing recombinant human S1P_1_ (circles, A and B) or human S1P_5_ (squares, C and D). Each concentration response curve is the mean ± standard error of the mean of 3–5 replicate experiments run in duplicate wells. S1P, sphingosine 1-phosphate; S1P_1_, sphingosine 1-phosphate receptor subtype 1; S1P_5_, sphingosine 1-phosphate receptor subtype 5.

Saturation binding analysis with [^3^H]-ozanimod yielded measurable specific binding to membranes expressing human S1P_1_ and S1P_5_ with determined dissociation constants of 0.63 nM and 3.13 nM, respectively (Figure 1). The observed specific binding to both S1P_1_ and S1P_5_ was completely displaced by the endogenous ligand, S1P, and all of the S1P receptor modulators assessed (Figure 2), which suggests that ozanimod itself binds within the orthosteric binding pocket and that all of the competitor compounds also bind to the same site. Binding affinities (K_i_ values) were calculated from the inhibition of [^3^H]-ozanimod binding curves using the Cheng-Prusoff equation (Cheng and Prusoff, 1973) (Table 1).

**TABLE 1:** Radioligand binding affinities for human S1P_1_ and S1P_5_.

Compound	Human S1P_1_ K_i_ (nM)	Human S1P_5_ K_i_ (nM)
Ozanimod	0.5 ± 0.03	1.93 ± 0.07
S1P	2.33 ± 0.17	2.69 ± 0.31
FTY720-p	0.1 ± 0.01	0.26 ± 0.03
Amiselimod-p	0.13 ± 0.02	0.25 ± 0.04
Ponesimod	9.37 ± 1.31	17.43 ± 4.32
Siponimod	0.74 ± 0.09	0.43 ± 0.08
KRP-203-p	0.22 ± 0.02	0.24 ± 0.04
Etrasimod	6.73 ± 0.62	16.3 ± 0.71

[^3^H]-ozanimod was competitively displaced with increasing concentration of test compound using Chinese hamster ovary cell membranes stable expressing recombinant human S1P_1_ or human S1P_5_. Data shown are the mean ± standard error of the mean of the binding affinities calculated for 3–5 replicate experiments run in duplicate wells.

S1P, sphingosine 1-phosphate; S1P_1_, sphingosine 1-phosphate receptor subtype 1; S1P_5_, sphingosine 1-phosphate receptor subtype 5.

### [^35^S]-GTPγS Binding

The potency and relative intrinsic activity of ozanimod and test S1P receptor modulators are shown in Table 2. All compounds tested displayed strong activity for S1P_1_, with potencies below 1 nM, except for ponesimod and etrasimod, which were 3.42 and 5.48 nM, respectively. The potency for human S1P_5_ was also similarly below 1 nM for all compounds except ozanimod, ponesimod, and etrasimod, in which it was approximately 10-fold weaker than that for S1P_1_. Notably, some partial agonism, or below 80% relative intrinsic activity, was observed at S1P_5_ with the phosphorylated compounds FTY720-p, amiselimod-p, and KRP-203-p, displaying 74%, 76.6%, and 45.6% activity, respectively. Also important to note is that the functional potencies correlated well with the binding affinities for both S1P_1_ and S1P_5_.

**TABLE 2:** [^35^S]-GTPγS binding potencies for human S1P_1_–S1P_5_.

Compound	Human S1P_1_		Human S1P_2_		Human S1P_3_		Human S1P_4_		Human S1P_5_	
	EC_50_ (nM)	IA (%)	EC_50_ (nM)	IA (%)	EC_50_ (nM)	IA (%)	EC_50_ (nM)	IA (%)	EC_50_ (nM)	IA (%)
Ozanimod	0.4 ± 0.03	85 ± 1	>10000	*34.8 ± 1*	>1111	89.4 ± 7.4	1486.6 ± 306.5	39.4 ± 5.3	5.84 ± 0.51	97 ± 3.7
FTY720-p	0.2 ± 0.01	84.9 ± 2.3	>1000	*33.2 ± 1*	1.33 ± 0.19	106.5 ± 8.3	2.06 ± 0.3	59.2 ± 5.4	0.49 ± 0.07	74 ± 5.1
Amiselimod-p	0.15 ± 0.01	84.1 ± 1.6	>4000	NR	18.98 ± 2.98	29.1 ± 1.6	2.23 ± 0.13	122.2 ± 3.6	0.58 ± 0.08	76.6 ± 6.9
Ponesimod	3.42 ± 1.17	85.8 ± 6.2	>10000	NR	89.52 ± 14.29	82.1 ± 7.5	>10000	*26.5 ± 2.5*	43.18 ± 11.99	92.9 ± 9.8
Siponimod	0.46 ± 0.05	82.1 ± 0.9	>10000	NR	>1111	90.7 ± 2.5	383.73 ± 67.82	81.5 ± 2.4	0.3 ± 0.02	102 ± 9.1
KRP-203-p	0.26 ± 0	85.7 ± 3.6	>10000	NR	3.17 ± 0.26	25.7 ± 3.8	3.13 ± 0.29	56.3 ± 3.8	0.69 ± 0.19	45.8 ± 7.6
Etrasimod	5.48 ± 0.46	86.6 ± 1.1	>10000	NR	1164.3 ± 354.1	81.2 ± 17	1125.2 ± 188.9	40.4 ± 4.6	58.87 ± 6.75	82 ± 4.4

[^35^S]-GTPγS binding in response to increasing concentrations of test compound performed using Chinese hamster ovary cell membranes stably expressing recombinant human S1P_1_–S1P_5_. Data shown are the mean ± standard error of the mean of the compound potencies as determined by the concentration required to elicit a half-maximal response (EC_50_) as well as the intrinsic activity (IA) relative to the maximal response generated with the endogenous ligand, S1P, which was taken to be 100%, calculated for 3–5 replicate experiments run in duplicate wells. NR indicates no response where the maximal response was less than 10% of that of S1P. Italic type indicates that the response was achieved at the top test compound concentration of 10000 nM, where no clear maximal response was defined.

[^35^S]-GTPγS, [^35^S]-guanosine-5'-(γ-thio)-triphosphate; S1P, sphingosine 1-phosphate; S1P_1_–S1P_5_, sphingosine 1-phosphate receptor subtypes 1 through 5.

When the activities of the S1P receptor modulators across all five S1P receptors were compared using 300 nM as a cutoff for potency, siponimod, ozanimod, and etrasimod were the most selective, demonstrating activity for only S1P_1_ and S1P_5_. Ponesimod was also selective for S1P_1_ and S1P_5_ but did display activity for S1P_3_ in the current study, with an EC_50_ of 89.52 nM. The phosphorylated compounds displayed the least selectivity, having potent activity for S1P_1_, S1P_3_, S1P_4_, and S1P_5_, with EC_50_ values ≤18.98 nM.

### S1P_1_-Mediated β-arrestin Recruitment

Since it is well documented that activation of S1P_1_ results in rapid receptor internalization away from the plasma membrane and into the intracellular compartment, the potency of the S1P modulators to induce the recruitment of β-arrestin was also measured. All of the test compounds activated β-arrestin recruitment; however, the potencies determined using the β-arrestin assay were less potent than for the GTPγS binding assay, with the exception of ponesimod, which is equipotent, and none of the test compounds elicited as robust a response as the endogenous ligand, S1P, with relative intrinsic activities calculate in the range of 61.2%–79.8% of the S1P response (Table 3). Notably, the binding K_i_ and GTPγS potencies are very closely aligned (within <2-fold of each other), with the exception again of ponesimod which appears to have weaker affinity compared with potency, and the β-arrestin potency was comparatively right shifted. This may be due to the fact that the radioligand binding and GTPγS binding assays were run in the same cell backgrounds, whereas β-arrestin recruitment was determined using a commercial assay kit and, hence, may have potentially different receptor expression levels. The β-arrestin data do however support the notion of functional antagonism for all S1P modulators through S1P_1_ activation. In addition, S1P_5_ does not internalize in response to agonists in our hands (data not shown) nor according to (Bigaud et al., 2018), and so β-arrestin assays were not conducted for this S1P receptor subtype.

**TABLE 3:** β-arrestin recruitment potency for human S1P_1_.

Compound	EC_50_ (nM)	Intrinsic Activity (%)
Ozanimod	1.12 ± 0.22	68.7 ± 3.5
FTY720-p	0.74 ± 0.06	79.8 ± 4.3
Amiselimod-p	2.83 ± 2.21	76.1 ± 11.6
Ponesimod	2.66 ± 0.42	61.2 ± 1.6
Siponimod	9.3 ± 2.79	68.6 ± 9.2
KRP-203-p	2.98 ± 0.96	72.3 ± 5.4
Etrasimod	11.31 ± 2.32	75.8 ± 1.8

Human S1P_1_ β -arrestin assay was carried out using PathHunter® eXpress EDG1 CHO-K1 β-arrestin GPCR Assay. Data shown are the mean ± standard error of the mean (*n* = 3–5) or standard deviation (*n* = 2) of the compound potencies as determined by the concentration required to elicit EC_50_ as well as the intrinsic activity relative to the maximal response generated with the endogenous ligand, S1P, which was taken to be 100%, calculated for 2–5 replicate experiments run in duplicate wells.

EC_50,_ half maximal response; S1P_1_, sphingosine 1-phosphate receptor subtype 1.

### Computational Chemistry

**FIGURE 3:**
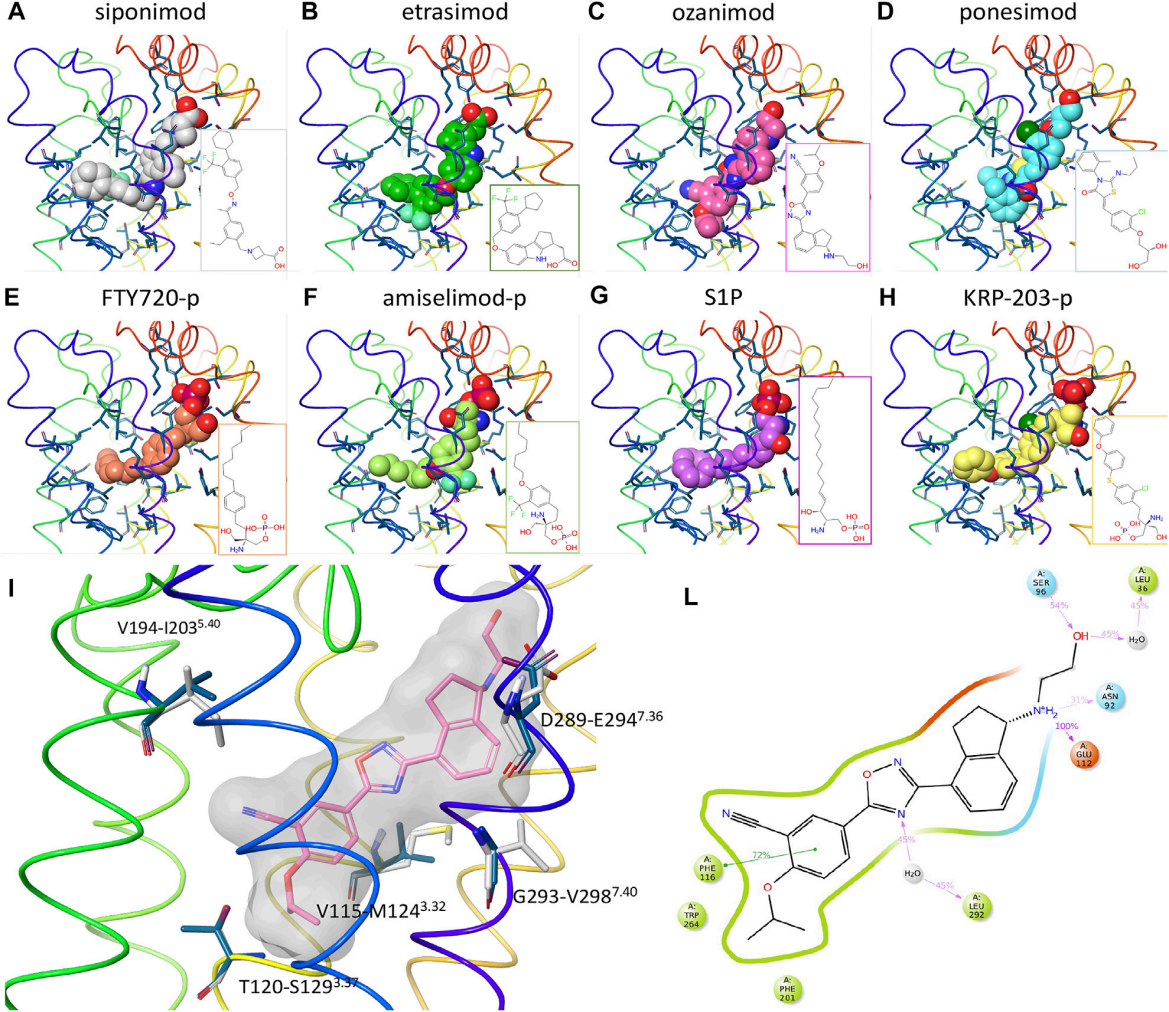
Computational chemistry analysis. **(A–H):** Predicted binding mode to S1P_5_ of siponimod **(A)**, etrasimod **(B)**, ozanimod **(C)**, ponesimod **(D)**, FTY720-p **(E)**, amiselimod-p **(F)**, S1P **(G)**, and KRP-203-p **(H)**. Ligands are included using the space-filling representation, and the orthosteric residues are shown as sticks with the backbone as a thin tube colored from red (N terminus) to purple (C terminus). Small-molecule two-dimensional (2D) structures are included in the bottom right part of the panels. **(I)** Predicted docked pose of ozanimod shown as sticks with pink carbon atoms and a grey transparent molecular surface. Residues close to the ligand and different between S1P_5_ (dark blue) and S1P_1_ (white) are included and labeled with the S1P_5_ residue numbers, followed by the S1P_1_ and family A G protein–coupled receptor generic numbering in superscript (Isberg et al., 2015). **(L):** 2D representation of ozanimod and close residues interacting with the ligand during the absolute free energy perturbation molecular dynamics (MD) simulation. Hydrophobic, polar, and negatively charged amino acids are shown in green, cyan, and red, respectively. Hydrogen bonds and ionic interactions are shown as magenta lines and π−π interactions as green lines. The amount of time in which the interactions are created during the MD simulation is shown as a percentage of the total simulation length (5 ns). S1P, sphingosine 1-phosphate; S1P_5_, sphingosine 1-phosphate receptor subtype 5.

To better understand the interactions of the different ligands with the receptor orthosteric site, a homology model of human S1P_5_ was created. After docking of ozanimod, the system was equilibrated using MD simulation in a fully explicit water-membrane environment. After the removal of ozanimod, the other small molecules were docked to the GPCR (Figures 3A–H) (Isberg et al., 2015). The ligands are predicted to bind to the same orthosteric site, creating similar interactions. Polar head groups of the ligands are proposed to establish a complex network of hydrogen-bonding interactions and salt bridges with the extracellular regions of transmembrane domain 1 (TM1), TM2, and TM7 and the N terminus of the receptor, where multiple charged and hydrophilic residues are present. The more hydrophobic tail of the ligands can bind deeper in the helical bundle extending toward TM3, TM5, TM6, and TM7 where several apolar amino acids are present. These interactions were predicted to be similar in both S1P_5_ and S1P_1_ and to result in high binding affinity and receptor activation. In particular, when the AFEP method was used on the proposed ligand-binding position, siponimod was predicted to have subnanomolar potency and be more potent for S1P_5_ than ozanimod, which is indeed what was determined with the experimental data (Supplementary Table S3). The MD simulation, part of the AFEP method, predicted strong and stable interactions between ozanimod’s protonated amino group and S1P_5_ Glu112 toward the extracellular portion of the receptor and good stacking interactions between Phe116 in the core of the orthosteric site and the phenyl ring of the ligand (Figure 3L) (Isberg et al., 2015).

## Discussion

In agreement with the radioligand binding data, when the computational chemistry tool is used, all of the small molecules are predicted to bind to the receptor orthosteric site. When evaluated using AFEP methods, the proposed binding modes of ozanimod and siponimod result in low-nanomolar binding affinity to S1P_1_ and S1P_5_, and indeed, all ligands were proposed to create similar strong interactions in both S1P_1_ and S1P_5_, explaining their potent activity in both binding affinity and GTPγS functional response (Supplementary Figures S1, S2). This is an important observation, because questions regarding the safe switching from one S1P receptor modulator to another are being asked with increasing frequency. The data presented here suggest that, due to the competitive nature of the ligand binding within both S1P_1_ and S1P_5_, there would not be an additive effect if more than one S1P receptor modulator compound were present at any given time. Instead, they would merely compete with each other, with the compound that has the highest exposure relative to its binding affinity having preferential occupancy of the binding site within either of the receptors. Since all the S1P modulators included in this study display similar receptor pharmacology and compete for binding at the same site, they can be considered interchangeable with one another. The choice of any one particular agent should therefore be made on the basis of overall therapeutic profile, and patients can be offered the opportunity to switch S1P medications without the potential concern of additive S1P pharmacology.

In relation to the observed dual activity of the modulators for both S1P_1_ and S1P_5_, there are five amino acids different between the two GPCRs in the orthosteric binding site and close to the ligand binding region in TM3, TM5, and TM7 (Figure 3I). In agreement with the comparable binding affinity and displacement data of the considered small molecules between S1P_1_ and S1P_5_, all five amino acids were predicted to have a limited effect on the binding affinity of the ligands. For example, and in support of these findings, the difference in free energy of binding of ozanimod between the 2 receptors predicted by the AFEP method was negligible (0.3 kcal/mol).

## Conclusion

To our knowledge, this is the first time a clear head-to-head comparison of binding affinity and functional potency has been assessed for the S1P receptor modulators currently approved for therapeutic use or in late stages of clinical development and hence may serve as a useful guide. It is interesting that the phosphorylated ligands demonstrated a clear trend to be the least selective and most potent but display more partial agonism for S1P_5_, and that the more recent novel small molecule compounds display more refined selectivity for S1P_1_ and S1P_5_. In conclusion, the experimentally derived competitive ligand binding modes for ozanimod, siponimod, etrasimod, ponesimod, FTY720-p, amiselimod-p, KRP-203-p, and S1P are in agreement with their computationally proposed competitive dual activity for S1P_1_ and S1P_5_.

## Data Availability

Details on restrictions and steps to access the datasets are available here: https://www.bms.com/researchers-and-partners/independent-research/data-sharing-request-process.html.
