# Supplementary material for: Competitive Binding of Ozanimod and Other Sphingosine 1-Phosphate Receptor Modulators at Receptor Subtypes 1 and 5
Source: Front Pharmacol. 2022 Jun 17;13:892097. doi: 10.3389/fphar.2022.892097 (PMC9247443; doi:10.3389/fphar.2022.892097)
Supplement: Supplementary file 1 [file DataSheet1.docx]

**Supplemental Material**

**Supplemental Table 1.** AFEP binding affinity predictions for siponimod and ozanimod.

| **Ligand** | **AFEP Predicted Free Energy of Binding (kcal/mol)** | |
| --- | --- | --- |
|  | **S1P_1_ Receptor** | **S1P_5_ Receptor** |
| Siponimod | -22.79 +/- 0.28 | -16.18 +/- 0.24 |
| Ozanimod | -14.30 +/- 0.18 | -14.64 +/- 0.21 |

AFEP predicted ligand-receptor free energy of binding in kcal/mol. AFEP, absolute free energy perturbation; S1P_1_, sphingosine 1-phosphate receptor subtype 1; S1P_5_, sphingosine 1-phosphate receptor subtype 5.

**Supplemental Figure 1.** Percentage of time the ligands are interacting with key S1P_1_ receptor residues during the molecular dynamics part of the AFEP method. H-bonds are in green, hydrophobic interactions in purple, ionic interaction in magenta, and water bridges in blue.

**Ozanimod in complex with S1P_1_ receptor**

**
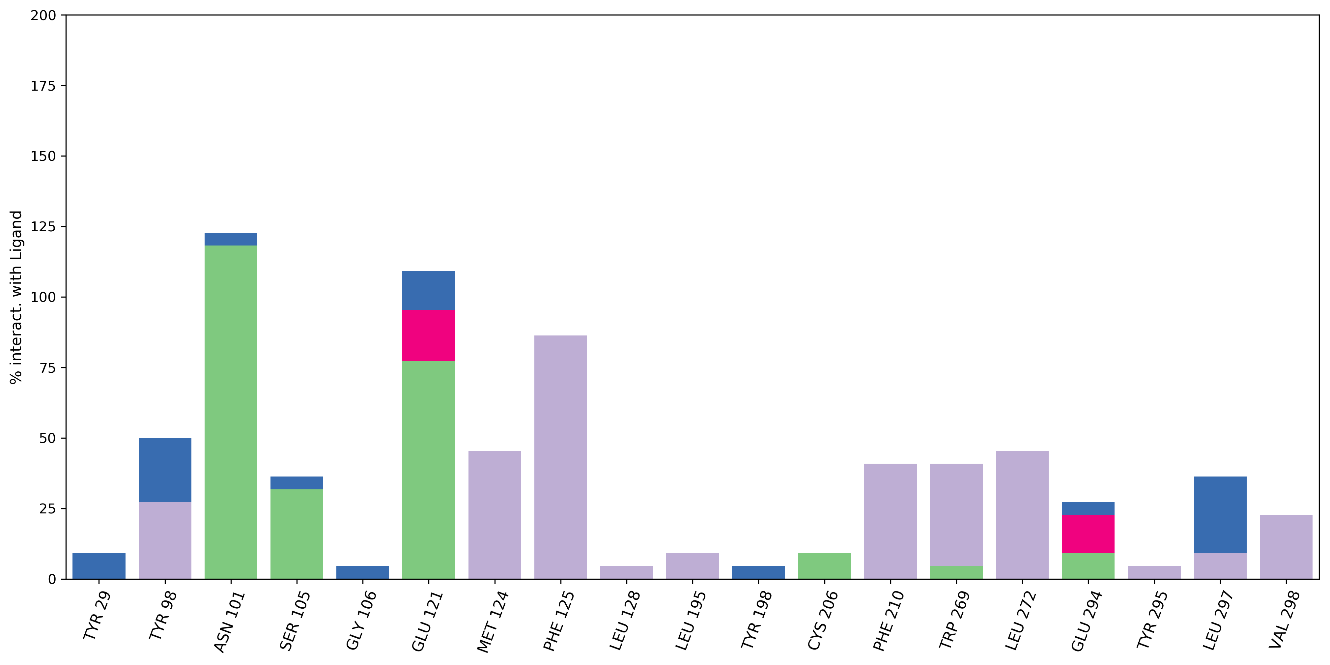
**

**Siponimod in complex with S1P_1_ receptor**


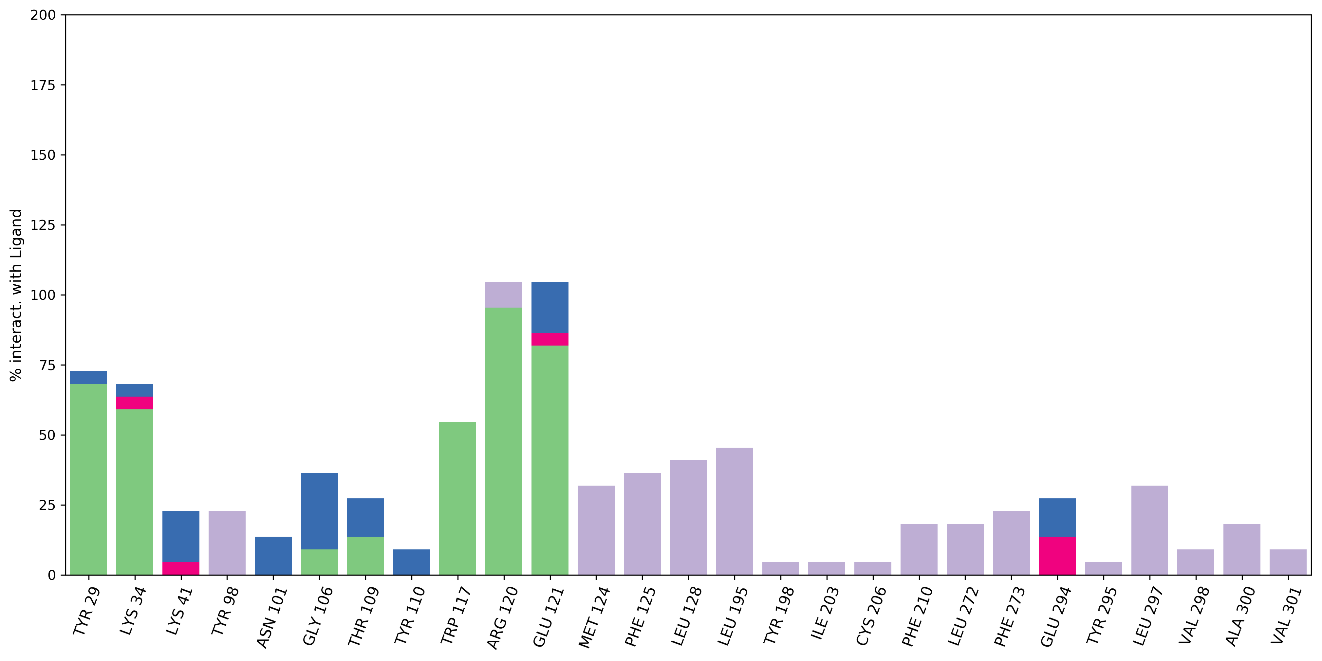


AFEP, absolute free energy perturbation; S1P_1_, sphingosine 1-phosphate receptor subtype 1.

**Supplemental Figure 2.** Percentage of time the ligands are interacting with key S1P_5_ receptor residues during the molecular dynamics part of the AFEP method. H-bonds are in green, hydrophobic interactions in purple, ionic interaction in magenta, and water bridges in blue.

**Ozanimod in complex with S1P_5_ receptor**


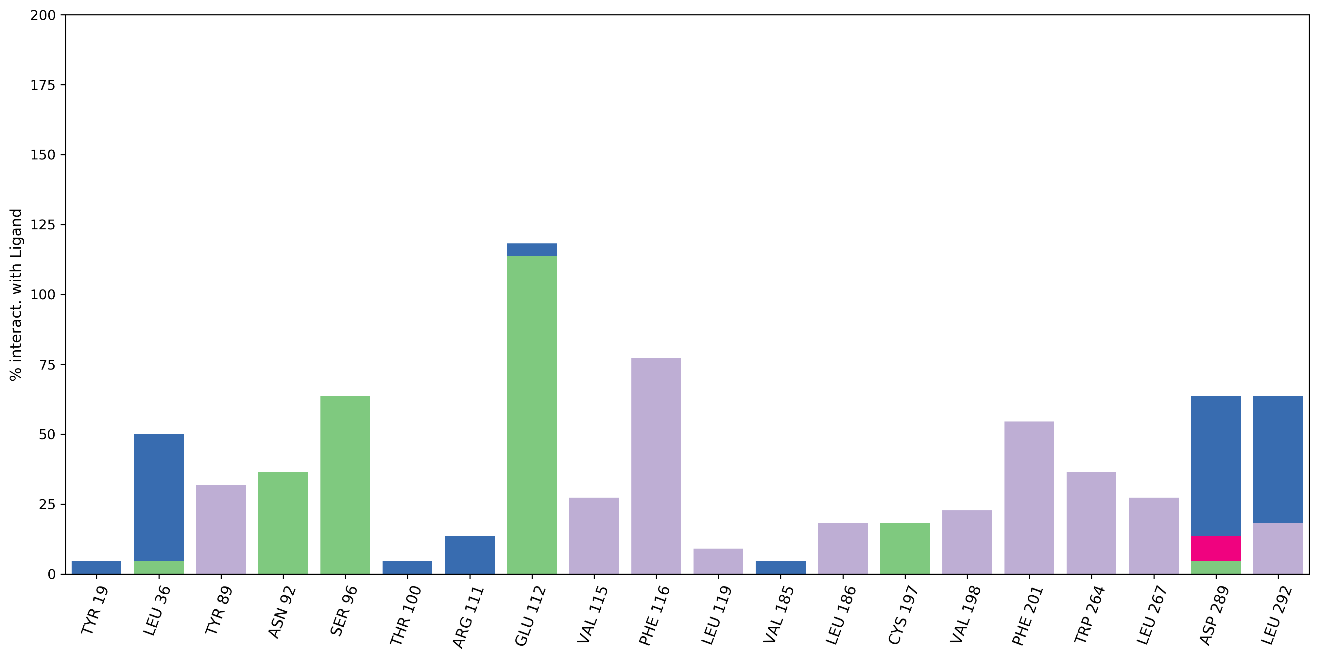


**Siponimod in complex with S1P_5_ receptor**


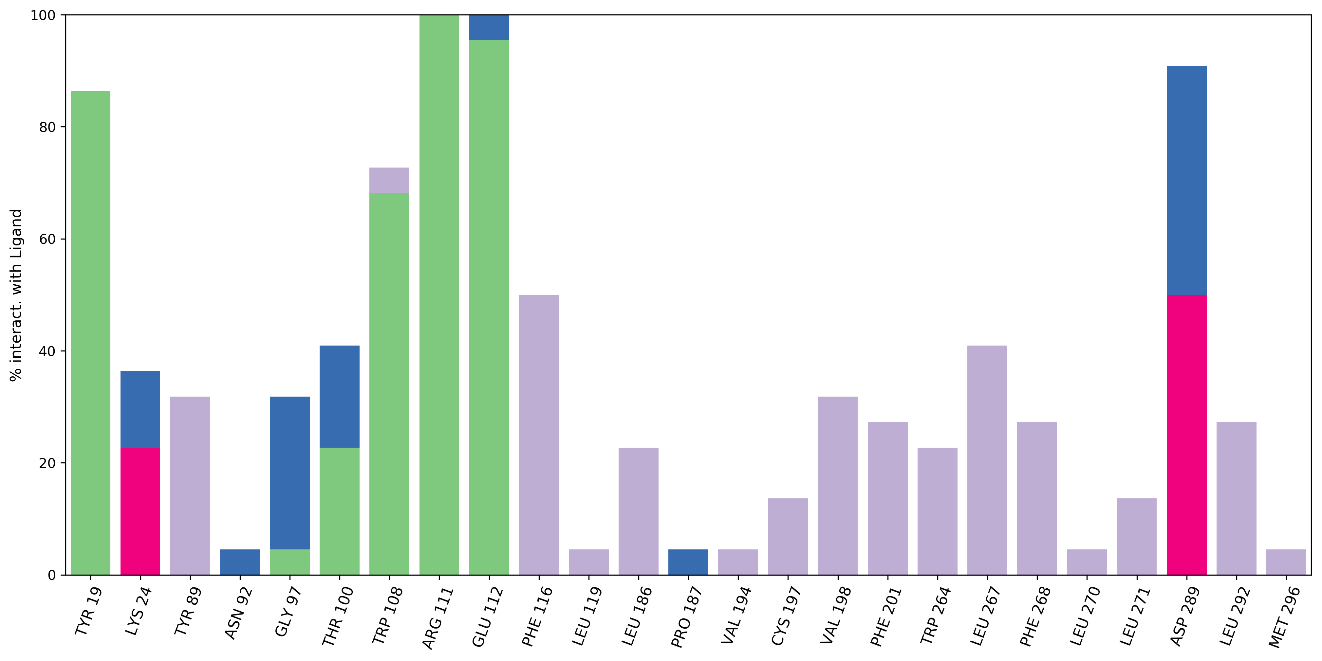


AFEP, absolute free energy perturbation; S1P_5_, sphingosine 1-phosphate receptor subtype 5.
